# 14K prolactin derived 14‐mer antiangiogenic peptide targets bradykinin‐/nitric oxide‐cGMP‐dependent angiogenesis

**DOI:** 10.1002/2211-5463.13895

**Published:** 2024-09-23

**Authors:** Jaeok Lee, Pavitra Kumar, Suganya Natarajan, So Hyeon Park, Syamantak Majumder, Lakshmikirupa Sundaresan, Kambadur Muralidhar, Jong‐Soon Choi, Hwa Jeong Lee, Suvro Chatterjee

**Affiliations:** ^1^ Pharmaceutical Laboratory, College of Pharmacy and Graduate School of Pharmaceutical Sciences Ewha Womans University Seoul Korea; ^2^ Vascular Biology Laboratory, AU‐KBS Research Centre Anna University, M.I.T. Campus Chennai India; ^3^ Hormone Research Laboratory, Department of Zoology University of Delhi India; ^4^ Division of Life Science Research Korea Basic Science Institute Daejeon Korea

**Keywords:** anti‐angiogenesis, antiangiogenic peptide, bradykinin, endothelial nitric oxide synthase, prolactin

## Abstract

Over the past few decades, VEGF‐targeted antiangiogenic therapy for cancers has gained increasing attention. Nevertheless, there are still several limitations such as the potential resistance mechanisms arising in cancer cells against these therapies and their potential adverse effects. These limitations highlight the need for novel anti‐angiogenesis molecules and better understanding of the mechanisms of tumor angiogenesis. In the present study, we investigated the antiangiogenic properties of a novel 14‐mer antiangiogenic peptide (14‐MAP) derived from N‐terminal 14 kDa buffalo prolactin and characterized its mode of action. 14‐MAP at the picomolar concentration inhibited VEGF‐ and bradykinin (an autacoid peptide expressed in vascular tissues in pathophysiology, BK)‐stimulated endothelial nitric oxide (eNO) production, cell migration, and proliferation in endothelial cells and vessel development in the chick embryo. Although this peptide inhibited both VEGF‐ and BK‐dependent angiogenic processes, its action was more pronounced in the latter. Moreover, the interference of 14‐MAP with the eNO synthase (eNOS)‐cyclic GMP pathway was also identified. A combination of a low dose of Avastin, a widely used drug targeting VEGF‐dependent angiogenesis, and 14‐MAP significantly reduced tumor size in an *in vivo* model of human colon cancer. Taken together, our results suggest that 14‐MAP, a BK‐ and eNOS‐dependent antiangiogenic peptide, might be useful for overcoming the limitation of VEGF‐targeted antiangiogenic therapy in cancer patients. However, further studies will be required to further characterize its mode of action and therapeutic potential.

## Graphical abstract

The study investigates a novel 14‐mer antiangiogenic peptide (14‐MAP) derived from buffalo prolactin, revealing its potent inhibitory effects on VEGF‐ and bradykinin‐induced angiogenesis. At picomolar concentrations, 14‐MAP disrupts endothelial nitric oxide production, cell migration, and blood vessel development.

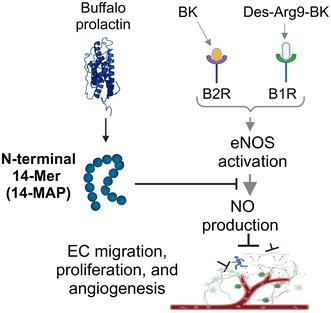

**Abbreviations**
14‐MAP: 14‐mer antiangiogenic peptide
BK: bradykinin
buPRL: buffalo prolactin
BUVEC: bovine umbilical vein endothelial cells
CAM: chick chorioallantoic membrane
DAF‐FM: diaminofluorescein‐FM diacetate
DMEM: Dulbecco's modified eagle medium
EC: endothelial cells
eNOS: endothelial nitric oxide synthase
FBS: fetal bovine serum
GMP: guanosine monophosphate
HUVEC: human umbilical vein endothelial cells
NO: nitric oxide
PBS: phosphate‐buffered saline
PI3K: phosphoinositide 3‐kinases
PKG: protein kinase
PRL: prolactin
RFU: relative fluorescence unit
SC: sildenafil citrate
Scr: scrambled
SEM: standard error mean
sGC: soluble guanylyl cyclase
VEGF: vascular endothelial growth factor
YC‐1: 3‐(5′‐hydroxymethyl‐2′‐furyl)‐1‐benzylindazole

## Introduction

Angiogenesis, the intricate process involving the formation of new blood vessels from preexisting vasculature, is pivotal for both physiological and pathological developments [1, 2, 3, 4]. Notably, in 1865, Rudolf Virchow observed a substantial vascular network in growing tumors—a finding that gained significant attention for the subsequent 150 years. This interest culminated when Judah Folkman introduced the revolutionary concept of anti‐angiogenesis therapy, specifically targeting and diminishing the effects of a critical growth factor, vascular endothelial growth factor (VEGF), to treat perfused tumors [5, 6]. Over the past few decades, extensive research focused on anti‐angiogenesis targeting VEGF in cancer and metastasis, setting the stage for clinical approvals [7]. Despite the clinical deployment of VEGF‐targeted antiangiogenic approaches, limitations persist. For instance, while Avastin (bevacizumab)—the first antiangiogenic drug—has garnered approval to treat metastatic colorectal cancer in combination with fluorouracil‐based chemotherapy for first‐ or second‐line treatment, its use post‐surgery remains unapproved [6, 8]. These limitations underscore the need for in‐depth mechanistic insights into anti‐angiogenesis therapies, necessitating exploration beyond VEGF and incorporating other hallmarks of tumors [6]. Addressing this need, our previous research introduced a 14 kDa (14K) buffalo prolactin (buPRL), which effectively antagonized VEGF‐ and bradykinin (BK)‐dependent endothelial nitric oxide (eNO) production—key processes for endothelial cell migration and tube formation [9]. Building on this, we synthesized a 14‐mer antiangiogenic peptide (14‐MAP) within the N‐terminal 14K buPRL sequence, aiming to leverage its potent antiangiogenic properties [9]. Bradykinin (BK), a classical 9‐mer peptide generated by the kallikrein‐kinin system, is implicated in several pathophysiological processes, including inflammation, cell proliferation, migration, coagulation, fibrogenesis, angiogenesis, and cancer [10, 11, 12, 13]. BK acts as an agonist for endothelial NO synthesis, promoting the sub‐cellular trafficking of eNOS in endothelial cells [14]. Additionally, BK receptors, particularly B1R and B2R, play significant roles in cancer growth and metastasis, with elevated expression observed in various cancers, including colon cancer [13, 14, 15, 16, 17, 18, 19]. Thus, targeting the BK‐receptor pathway presents a promising avenue for cancer therapy.

The endothelial NO synthase (eNOS) pathway is crucial for angiogenesis, vascular permeability, and relaxation—stimulated by vasoactive factors such as VEGF and BK. eNOS‐produced NO correlates positively with endothelial cell migration [20, 21]. Our research indicates that disruptions in the NO pathway impact vasculogenesis [4], angiogenesis [22], organ development [23], and embryogenesis [24, 25]. The vascular eNOS/NO/soluble guanylate cyclase (sGC)/cyclic GMP (cGMP)/cGMP‐dependent protein kinase (PKG) pathway is activated through PI3K/Akt signaling in the endothelium [26]. Gonzalez *et al*. [27] demonstrated that the N‐terminal 16K human PRL (hPRL) inhibits eNOS activation mediated by VEGF, BK, and acetylcholine (Ach) in bovine and human umbilical vein endothelial cells (BUVEC and HUVEC). Our studies corroborated these findings, noting that 14K buPRL specifically inhibited eNO production in immortalized EAhy926 cells via VEGF and BK pathways, but not through Ach [9]. BK, as an eNOS agonist, plays a pivotal role in the sub‐cellular localization of eNOS, implicating the BK‐PI3K‐Calcium calmodulin‐eNOS signaling pathway [28].

We demonstrated that 14‐MAP exhibits potent inhibitory effects on BK‐induced endothelial functions, including NO production, cell migration, and chick embryonic vessel formation, even at picomolar concentrations. Mechanistically, the antiangiogenic properties of the peptide were elucidated, and its additional effect in reducing human colon tumor size when co‐administered with Avastin was validated. This study suggests that 14‐MAP holds potential as an anti‐tumor drug, pending further molecular investigations including to clarify BK‐/NO‐cGMP‐dependent antiangiogenic effect by 14‐MAP on tumor reduction.

## Materials and methods

### Materials

The immortalized human endothelial cell line, EAhy926, and the human colon adenocarcinoma cell line, HT29, were gifted from C. J. S. Edgel (University of North Carolina, USA) and from U. Schumacher (University of Medical Center Hamburg‐Eppendorf, Germany), respectively. Brown leghorn (*Gallus gallus*) fertilized eggs were purchased from a poultry farm (Potheri, Chennai, India).

14‐MAP (AQGKGFITMALNSC (pI 8.27/molecular weight (M.W.) approx.1.4 kDa) and 14‐MAP scramble (Scr) (GSQCAAGTMNLKIF)) were gifted from J. K. Bang (Korea Basic Science Institute (KBSI), Korea). BK, VEGF, des‐Arg9‐BK (B1R agonist), des‐Arg9‐Leu8]‐BK (B1R antagonist), and 3‐(5′‐Hydroxymethyl‐2′‐furyl)‐1‐benzyl indazole were purchased from Sigma‐Aldrich (St. Louis, MO, USA). 8‐Bromoguanosine 3′, 5′‐cyclic monophosphate and DAF‐FM were purchased from Calbiochem (Darmstadt, Germany) and Molecular Probes® (Thermo Fisher Scientific, Rockford, IL, USA), respectively. Avastin was obtained from Selleckchem (Houston, TX, USA). SpermineNONOate was purchased from Cayman Chemicals (Ann Arbor, MI, USA). All other reagents were of molecular or cell culture grade.

### Cell culture

EAhy926 cells [29] and HT29 cells were cultured in DMEM and RPMI1640, respectively, supplemented with 10% fetal bovine serum and penicillin (100 U·mL^−1^)/streptomycin (50 pg·mL^−1^) at 37 °C and 5% CO_2_ as described [30].

### Cell viability assay

EAhy926 cells were seeded in 24‐well plates and incubated at 37 °C and 5% CO_2_ to achieve 60–70% confluence. The cells were treated with different concentrations of 14‐MAP peptide ranging from 1 pg·mL^−1^ to 10 μg·mL^−1^ for 4 h (the predicted half‐life [31]). After treatment period, trypan blue (0.4 mg·mL^−1^) was added to the media and incubated further for 15 min. After removing media, cells were washed once with PBS and further PBS was added to the wells. The number of cells with a blue nucleus was counted manually under the 4× objective of on Olympus inverted microscope.

### Scrape wound healing assay

The endothelial cells were cultured as a monolayer in a 96‐well plate up to 90% confluence. One straight‐line scratch was made across each well using a sterile p10 pipet tip as described [32]. Debris from the scratch area was washed with media. Cells were treated with the media with 10% FBS containing the respective treatment and incubated. Bright‐field images were taken under 4× magnification with a camera adapted to the Olympus XL70 bright field microscope at every 2 h interval. HT29 cells were cultured as a monolayer in a 24‐well plate up to 85–90% confluence. Cells were treated with the media without serum, containing the respective treatment. Images were taken under 5× magnification with a camera adapted to Zeiss AXIO observer 7 inverted microscope (Axio observer 7; Car Zeiss, Jena, Germany) equipped at the Drug Development Research Core Center in Ewha Womans University, at every 24 h intervals. Finally, the percentage of wound healing was quantified from the images using imagej software [33]. The data were collected and plotted from a minimum of three independent experiments. The assay was normalized for cell number, viability, and proliferation index.

### NO production assay

Nitric oxide was measured using a DAF‐FM fluorescence probe [34]. EAhy926 cells were seeded in a 96‐well plate and incubated overnight to obtain 90–95% confluence. The media was replaced with appropriate treatment media and incubated for 2 h in a CO_2_ incubator. After incubation, the media was removed, cells were washed with PBS, and 100 μL of DAF‐FM (3 μm) in PBS was added to each well. The plate was incubated for 20 min in the dark. One hundred microliter of supernatant from cells was collected and transferred to a fresh 96‐well plate. Then, absorbance was read at 495/515 nm using a fluorescence spectrophotometer (Cary Eclipse; Agilent Technology, Bangalore, India). The data were collected and plotted from a minimum of three independent experiments.

### Cell proliferation assay

The endothelial cells were seeded in a 96‐well plate around 15–20% confluency and incubated for 24 h. After starvation for 1–18 h, cells were treated with each factor with 0.2% serum medium, and then, an MTT assay [35] was performed on the cells 48 h later. The absorbance was read at 540 nm using a microplate reader (680; Bio‐Rad (Hercules, CA, USA)). Cell viability was expressed as a percentage of cell proliferation. The data were collected and plotted from a minimum of three independent experiments.

HT29 cells were seeded in a 96‐well plate around 15–20% confluency and incubated for 24 h. Cells were treated with each factor without serum medium, and then, sulforhodamine B staining assay was performed on the cells 24 and 48 h later. The data were collected and plotted from a minimum of three independent experiments.

### Chick chorioallantoic membrane assay

Chick chorioallantoic membrane (CAM) assay was performed with fertilized eggs at Hamburger‐Hamilton's stage 16 of chick development [36]. The eggs were incubated at 37 °C in a humidified incubator (Southern Incubators, Chennai, India) for 3 days and on the fourth day opened north polar under sterile conditions. Whatman filter paper disks contacting target factors including control were placed over the vasculature of area vasculosa, and then, the eggs were incubated for 8 h. The vasculature was imaged every 2 h from the time of drug treatment. All images were quantified using the angio‐quant software [37] and total vessel length, number of junctions, and vessel size were calculated. The percentage change in these parameters was calculated and plotted against the treatment. The data were collected and plotted from a minimum of three independent experiments.

### Dose determination of 14‐MAP and avastin *in vivo*

HT29 cells (5 × 10^6^ cells/100 μL PBS and Matrigel® mixture) were subcutaneously inoculated on the flank of balb/c nude mice (5‐week‐old (16.3–19.5 g), female). When tumor volume was over 100 mm^3^, different doses of Avastin (0.05, 0.5, and 5 mg·kg^−1^) and 14‐MAP (0.5 and 1 μg·kg^−1^) versus control (PBS) were given twice per week for three consecutive weeks by an intravenous injection to the tail vein (*n* = 3–4/group). The tumor size was measured with a digital caliper, twice a week. The mice were euthanized when the tumor volume reached 2000 mm^3^ or if there was any distress before. All animal care and experimentation followed the procedure approved by the Ewha Womans University Institutional Animal Care and Use Committee (IACUC) (no. 2016‐16‐062), Korea.

### Xenograft nude mice experiment

HT29 (3.5–5.0 × 10^6^ cells/100 μL PBS and Matrigel® mixture) was subcutaneously inoculated on the flank of Balb/c nude mice (5‐week‐old (18.2–22.5 g), male). When tumor volume was approximately 100 mm^3^, the treatments of 14‐MAP, Avastin, and the combination of both versus control (PBS) were performed every 3 days for 3 weeks by an intravenous injection to the tail vein (*n* = 5/group). The body weight was monitored, and tumor size was measured with a digital caliper twice a week. The tumor volume was calculated using the formula (length × width^2^)/2. All animal care and experimentation followed the procedure approved by the Ewha Womans University Institutional Animal Care and Use Committee (no. 2016‐062), Korea.

### Animal toxicity study

ICR mice (6‐week‐old (27.7–34.4 g), male) were treated with four different doses of 14‐MAP (0.01, 0.1, 0.5 and 1 μg·kg^−1^) and PBS (control) every day for 8 days by an intravenous injection to the tail vein (*n* = 5/each group). The body weight was monitored twice a week for an initial 2 weeks and then it was done once a week for the last 2 weeks. The mice were checked daily for their condition such as behavior, mortality, etc. Four weeks later, the organs such as a liver, stomach, intestines, spleen, kidney, lung, heart, etc. were ascertained in all mice. All animal care and experimentation followed the procedure approved by the Ewha Womans University Institutional Animal Care and Use Committee (IACUC) (no. 2016‐062), Korea.

### Colony formation assay

HT29 and MCF‐7 cells (200/well, 24‐well plate) were seeded as a single cell. After 24 h, 14‐MAP (50 pg·mL^−1^), Avastin (5 ng·mL^−1^), and the combination vs control were added to the cells, and the cells were incubated to form colonies for 8–13 days. The colonies were stained with 0.5% crystal violet after methanol fixation.

### Statistical analysis

Statistical analysis was conducted using Tukey's or Dunnett's test in conjunction with the one‐way ANOVA test or Dunnett's T3 test in conjunction with Welch's one‐way ANOVA. For *in vivo* experiments, the Student's *t*‐test and nonparametric Kruskal–Wallis test were performed. graphpad prism was used for the analysis (version 8.0.0 (224), La Jolla, CA, USA). All mean data were represented with standard error (SEM). The *P* value < 0.05 was set as a statistical significance.

## Results

### 14‐MAP inhibits VEGF‐ and BK‐induced EC functions

**Fig. 1:**
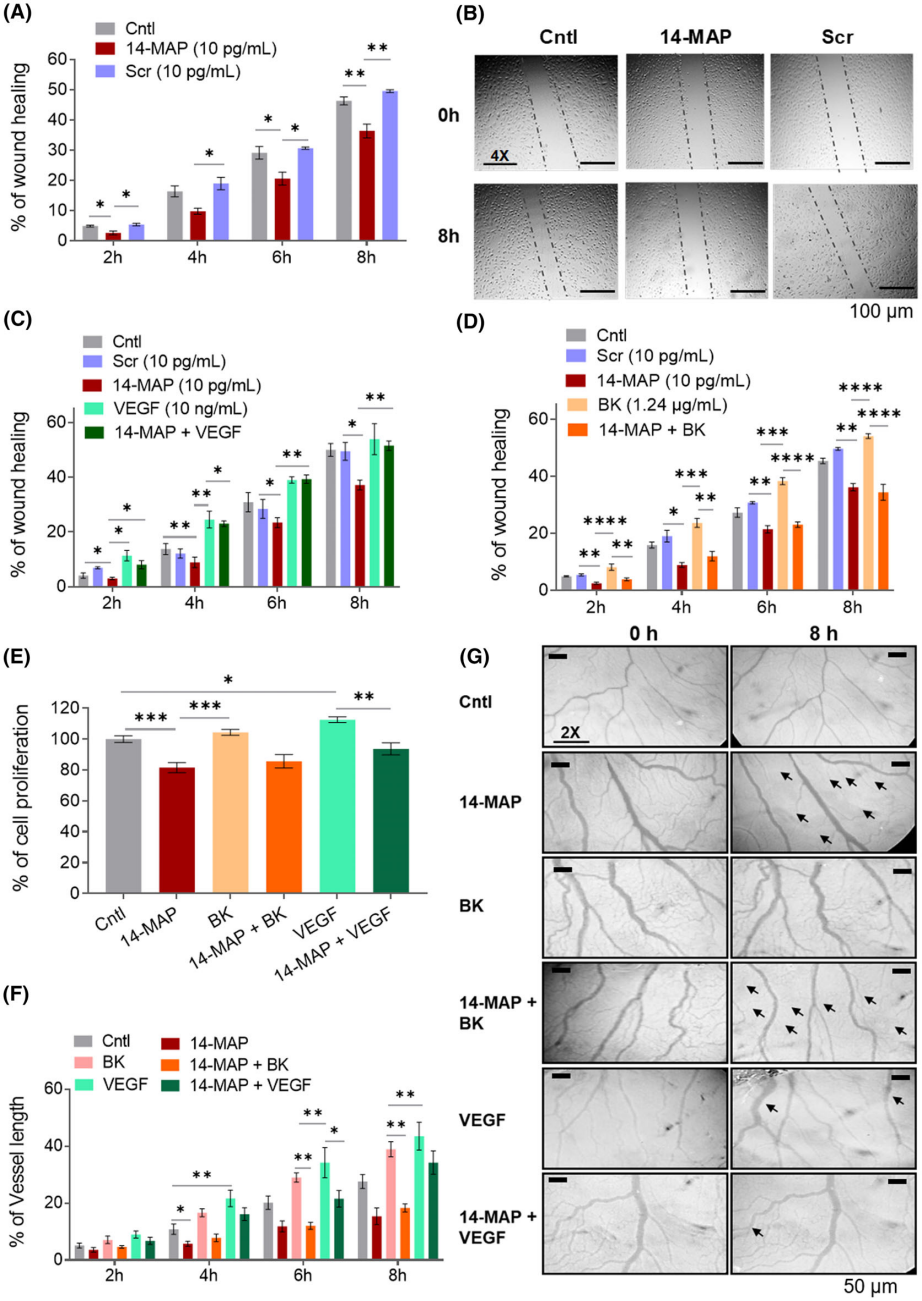
Inhibition of VEGF‐ and BK‐induced endothelial cell functions by 14‐MAP. (A, B) Cell migration analysis by wound healing assay. Cell migration analysis by wound healing assay of 14‐MAP on VEGF pathway, scale 100 μm (C) and BK pathway (D). (E) Cell proliferation analysis, and (F, G) (scale 50 μm) Angiogenic vessel growth analysis by CAM assay of 14‐MAP on VEGF‐ and BK‐pathways, respectively. 14‐MAP and Scr, 10 pg·mL^−1^ (approx. 7 pm); VEGF, 10 ng·mL^−1^ (approx. 0.3 nm); BK, 1.24 μg·mL^−1^ (approx. 1.24 μm). Cntl, control; Scr, scramble (GSQCAAGTMNLKIF). *P < 0.05; **P < 0.01; ***P < 0.001; ****P < 0.0001. Arrows indicate the deteriorating (14‐MAP, 14‐MAP+BK, and 14‐MAP+VEGF) and ameliorating (VEGF) vascular network. Statistical analysis was conducted using Tukey's or Dunnett's test in conjunction with the one‐way ANOVA test or Dunnett's T3 test in conjunction with Welch's one‐way ANOVA. All mean data were represented with standard error (SEM). Each experiment represents three independent biological experiments.

The antiangiogenic effect of the 14‐MAP peptide was confirmed by wound healing analysis (Fig. 1A,B). Excessively low concentration of the 14‐MAP peptide sufficiently inhibited EC migration. The significant inhibition was progressive in a time‐dependent manner, compared to the control (*P* < 0.05–0.01). Scr peptide did not show any difference from the control. The inhibitory effect was validated in chick embryo vessel development (Fig. S1). The working concentration of 14‐MAP was determined in the range of pico‐molarity and had no cytotoxicity (Fig. S2).

To understand the 14‐MAP‐mediated antiangiogenic pathway, we investigated the effects of the peptide on VEGF‐ and BK‐induced EC functions, confirmed as related pathways to the inhibition of 14K buPRL in our previous study [9]. 14‐MAP inhibited the cell migration stimulated by exogenous VEGF and BK as well as endogenous EC migration significantly (*P* < 0.05) (Fig. 1C,D). The inhibition value of 14‐MAP on the exogenous BK‐increased EC migration developed in a time‐dependent manner (*P* < 0.01–0.0001) and was similar to that of the peptide on the endogenous EC migration (*P* < 0.05) (Fig. 1D), whereas the inhibition level of 14‐MAP on the exogenous VEGF‐increased EC migration was just similar with the control level (Fig. 1C). Scr peptide did not significantly affect BK‐induced EC migration (Fig. S3A).

Moreover, the inhibitory effect of 14‐MAP on VEGF‐ and BK‐increased EC proliferation (for 48 h) was investigated (Fig. 1E). 14‐MAP significantly inhibited EC proliferation up to approximately 20% compared to the control (*P* < 0.001). Exogenous VEGF‐ and BK‐induced EC proliferation appeared to increase by 5% and 10%, respectively, compared to the control. The exogenously stimulated EC proliferations were significantly inhibited by 14‐MAP (*P* < 0.001 and *P* < 0.01, respectively) as much as endogenous inhibition (approximately 20%) by the peptide. Scr did not significantly affect EC proliferation or VEGF‐ and BK‐induced proliferation, either (Fig. S3B).

14‐MAP inhibition of VEGF‐ and BK‐induced EC functions estimated by an *in vitro* system was confirmed by an *ex vivo* system. The embryo vessel development enhanced by VEGF or BK was inhibited by the antiangiogenic peptide, 14‐MAP (Fig. 1F,G, Fig. S3C,D). BK‐enhanced vessel length development was decreased by 14‐MAP (*P* < 0.01) as much as 14‐MAP‐decreased vessel length development (*P* < 0.05), while VEGF‐enhanced vessel length development (*P* < 0.05–0.01) was decreased by 14‐MAP as much as the control level (Fig. 1F,G). These patterns were also monitored in the development of embryo vessel size and junctions (Fig. S3C,D). These results of *in vitro* and *ex vivo* tests indicate that the antiangiogenic peptide 14‐MAP is more correlated to the BK pathway than the VEGF pathway on EC functions.

### 14‐MAP interferes with eNOS pathway on EC migration

**Fig. 2:**
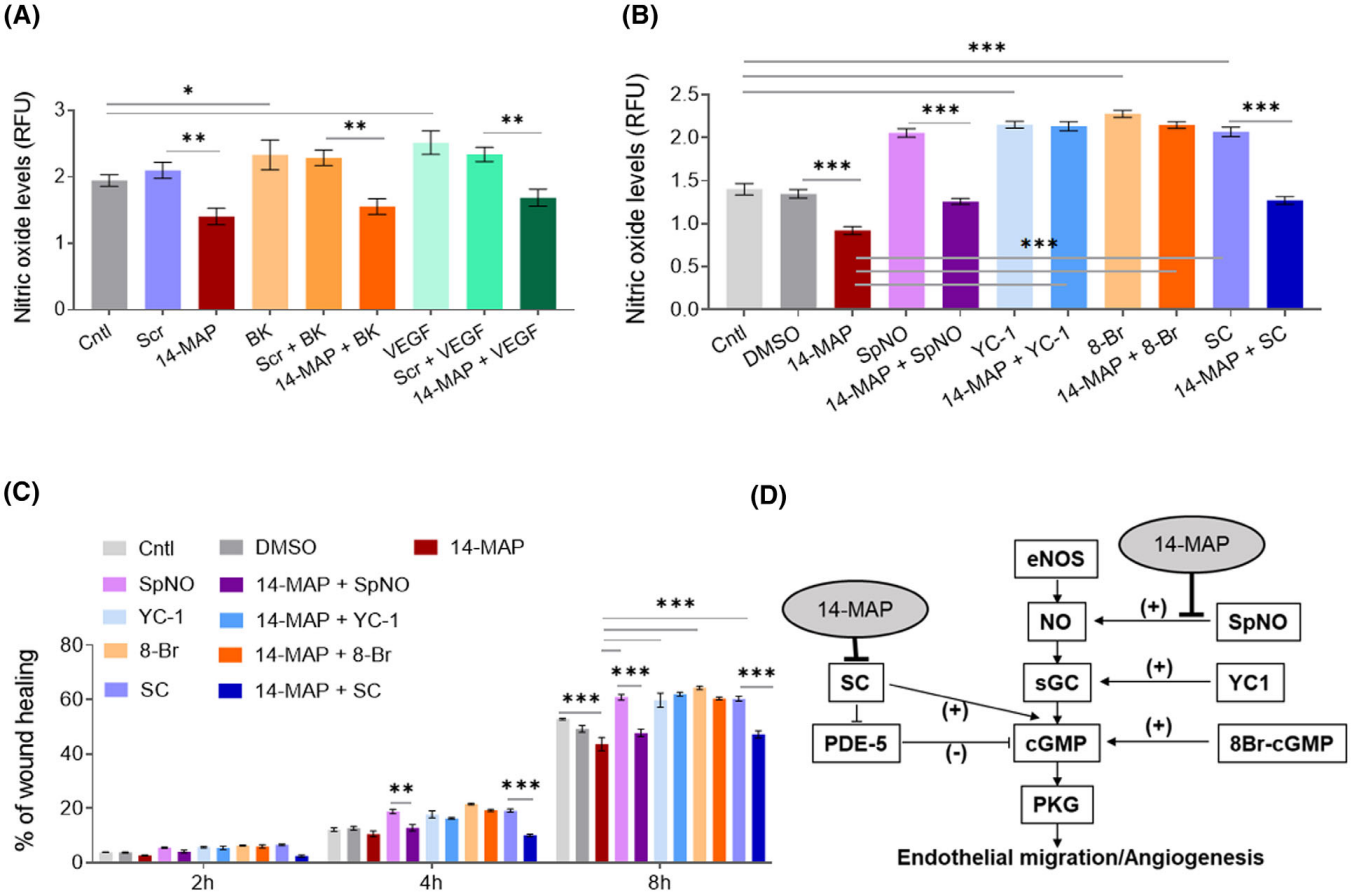
Inhibitory effect of 14‐MAP in eNOS pathway. (A) NO production analysis of 14‐MAP on VEGF pathway in EC. (B) NO production analysis of 14‐MAP on eNOS/NO/sGC/cGMP pathway in EC. (C) Cell migration analysis of 14‐MAP on eNOS/NO/sGC/cGMP pathway in EC. (D) Diagram of eNOS/NO/sGC/cGMP pathway correlated with 14‐MAP. Cntl, control; Scr, scramble (GSQCAAGTMNKIF). **P < 0.01; ***P < 0.001; ****P < 0.0001. Statistical analysis was conducted using Tukey's or Dunnett's test in conjunction with the one‐way ANOVA test or Dunnett's T3 test in conjunction with Welch's one‐way ANOVA. All mean data were represented with standard error (SEM). Each experiment represents three independent biological experiments.

To reveal the mechanism of 14‐MAP interference on angiogenesis, we investigated the effect of 14‐MAP on the eNOS pathway. 14‐MAP‐mediated NO inhibition was confirmed on endogenous NO production in EC (approximately 0.5 RFU value decrease (*P* < 0.01) *vs* control) (Fig. 2A). Moreover, the peptide decreased exogenous BK‐ and VEGF‐enhanced NO production (0.8 decrease (*P* < 0.001) and 0.8 decrease (*P* < 0.001), respectively) (Fig. 2A). Scr peptide did not induce any significant changes in the NO production increased by BK and VEGF as well as in the NO level of control.

To comprehend what step in the pathway is interfered by 14‐MAP on EC migration, we also studied NO production in EC (Fig. 2B) and EC migration (Fig. 2C) after co‐treatment of 14‐MAP with each inducer, SpNO (NO donor) [38], YC1 (soluble guanylate cyclase inducer) [39], 8Br‐cGMP (cGMP analog) [40] and SC (cGMP stabilizer) [41], on the eNOS/NO/sGC/cGMP pathway (Fig. 2D). DMSO used for dissolving the NO inducers did not affect NO production and EC migration (Fig. 2B,C). 14‐MAP significantly inhibited NO production increased by SpNO (0.7, *P* < 0.001) and SC (0.8, *P* < 0.001), but not by YC1 and 8Br‐cGMP (Fig. 2B). The inhibited level by 14‐MAP on the SpNO‐ and SC‐increased NO was as much as the NO production of the control. This pattern was confirmed on EC migration (Fig. 2C). 14‐MAP inhibited EC migration induced by SpNO (13.1% at 8 h, *P* < 0.001) and SC (13.1% at 8 h, *P* < 0.001) time‐dependently, but no differences were monitored on YC‐ and 8 Br‐cGMP‐induced EC migration when co‐treated with 14‐MAP (Fig. 2C). Therefore, it was observed that SpNO‐ and SC‐induced NO production and EC migration were correlated with 14‐MAP inhibition (Fig. 2D).

### 14‐MAP interferes with BK‐BK receptor pathway on EC functions

**Fig. 3:**
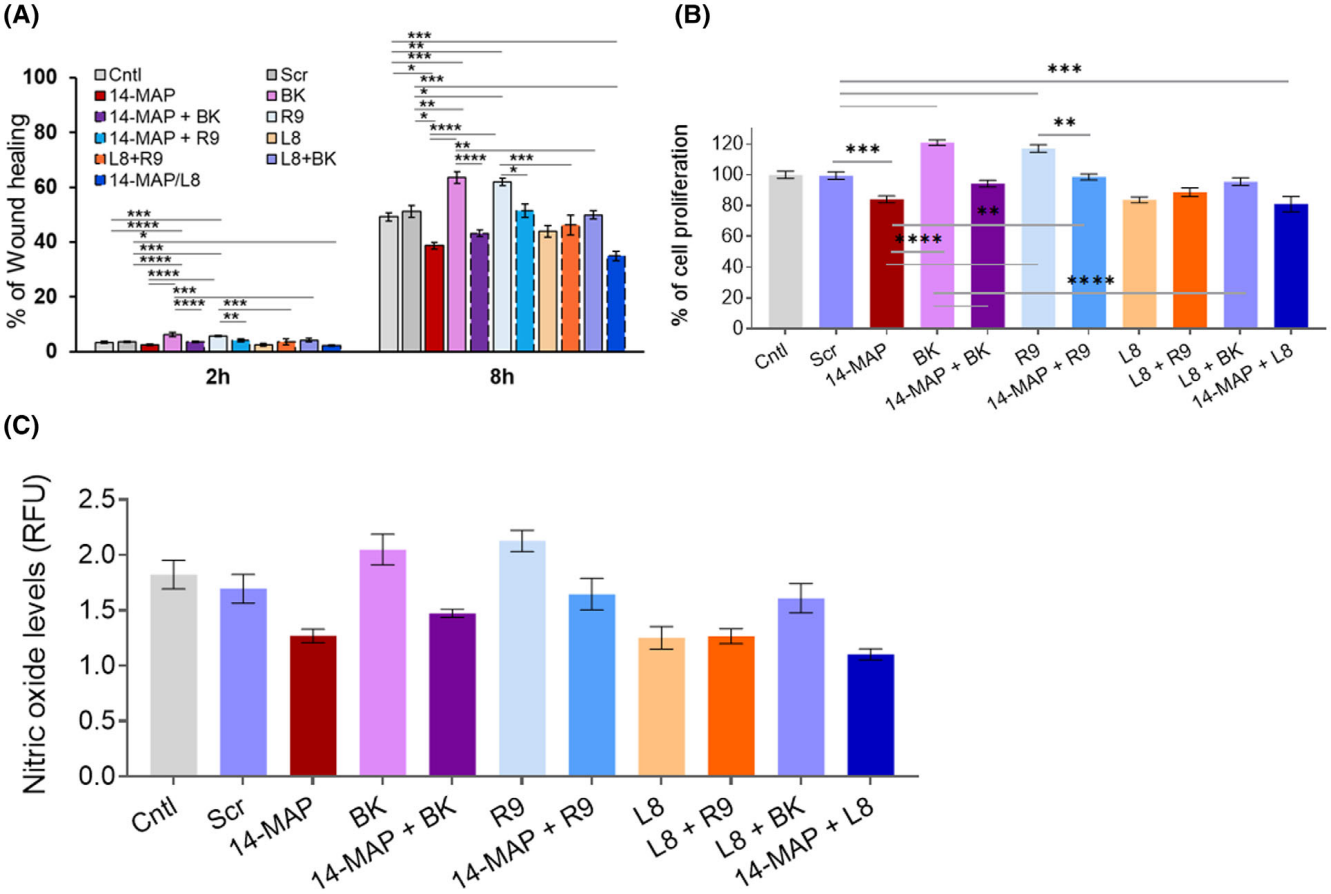
Interference of BK‐BKRs pathway by 14‐MAP. (A) EC cell migration. (B) EC proliferation. (C) NO production. 14, 14‐MAP; BK, B2R agonist; Cntl, control; L8, [Leu8]Des‐Arg9‐BK/B1R antagonist; R9, Des‐Arg9‐BK/B1R agonist; Scr, scramble. *P < 0.05; **P < 0.01; ***P < 0.001; ****P < 0.0001. Statistical analysis was conducted using Tukey's or Dunnett's test in conjunction with the one‐way ANOVA test or Dunnett's T3 test in conjunction with Welch's one‐way ANOVA. All mean data were represented with standard error (SEM). Each experiment represents three independent biological experiments.

Meanwhile, we considered to the correlation between the 14‐MAP and BK pathway (Fig. 1D,F,G). To understand which BK receptor (B1R or B2R) (Fig. S4) was related to 14‐MAP, we investigated the effect of 14‐MAP on BK‐B1R and ‐B2R pathways by EC cell migration, proliferation, and NO production (Fig. 3). When 14‐MAP was treated with an agonist of B1R and B2R in EC, the peptide significantly inhibited Des‐Arg9‐BK (R9, B1R agonist)‐ and BK (B2R agonist)‐increased EC migrations (12% (*P* < 0.05) and 21% (*P* < 0.0001) at 8 h, respectively) as well as an endogenous EC migration (12% (*P* < 0.05) at 8 h) (Fig. 3A). B1R antagonist [Leu8]Des‐Arg9‐BK (L8) also inhibited R9‐ and BK‐increased EC migrations (17% (*P* < 0.001) and 13% (*P* < 0.01) at 8 h, respectively) as well as endogenous EC migration (8% at 8 h) (Fig. 3A). In addition, the combination treatment of 14‐MAP with L8 induced a similar level of inhibition as by 14‐MAP alone, compared to control (14% (*P* < 0.001) at 8 h). The level of 14‐MAP inhibition of the B2R agonist (BK)/B2R pathway was the highest, and the level of 14‐MAP inhibition of the B1R agonist (R9)/B1R pathway was similar to the level of B1R antagonist (L8) inhibition of the B2R agonist/B2R pathway. Therefore, the inhibition effect of 14‐MAP showed more interference with BK/B2R pathway‐induced EC migration.

This pattern was also confirmed in EC proliferation (Fig. 3B) and eNO production (Fig. 3C). 14‐MAP significantly inhibited exogenous BK‐ and R9‐induced EC proliferation increase (24% (*P* < 0.0001) and 18% (*P* < 0.01), respectively) as well as endogenous EC proliferation (16%, *P* < 0.001), and L8 inhibited exogenous BK‐ and R9‐increased EC proliferation (25% (*P* < 0.0001) and 28% (*P* < 0.0001), respectively) as well as endogenous EC proliferation by L8 (16%, *P* < 0.001) (Fig. 3B). The combination treatment of 14‐MAP with L8 was similar to the inhibition level of 14‐MAP alone or L8 alone, compared to control (14%, *P* < 0.001). The inhibition of BK‐ and R9‐increased eNO production as well as endogenous eNO production by 14‐MAP (0.6%, 0.5%, and 0.5%, respectively), and BK‐ and R9‐increased eNO productions, as well as endogenous eNO production by L8 (0.5%, 0.8% and 0.6%, respectively), was confirmed (Fig. 3C). The combination treatment of 14‐MAP with L8 was slightly higher than the inhibition level of 14‐MAP alone or L8 alone compared to control (0.7%) (Fig. 3C). Finally, these results led to the idea that 14‐MAP‐mediated inhibition was related to the BK‐receptor pathway and more interfered with the BK/B2R pathway than the R9/B1R pathway on EC functions.

### The antiangiogenic peptide 14‐MAP does not influence *in vivo* growth of human colon cancer

**Fig. 4:**
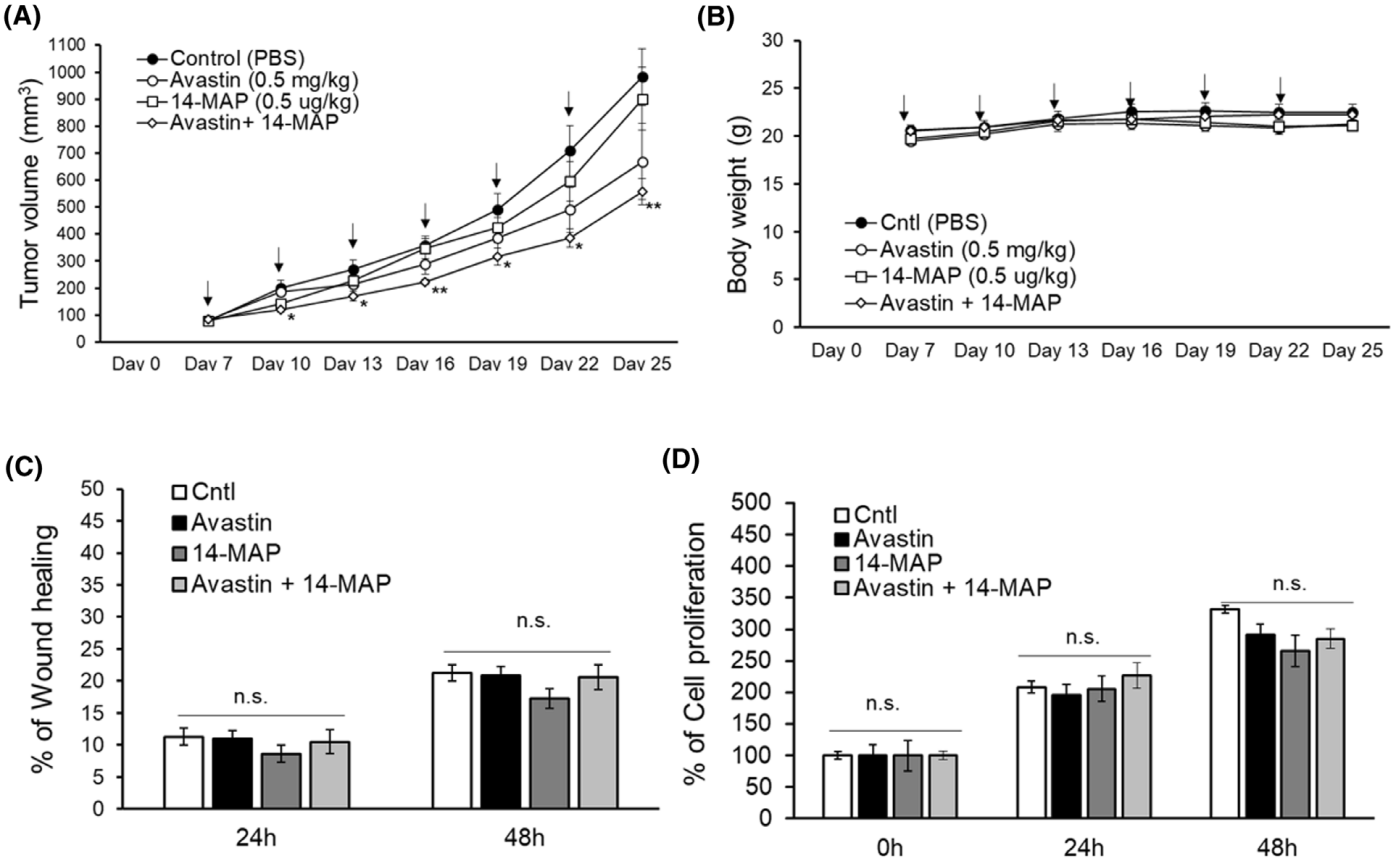
14‐MAP effects on cancer. (A) Tumor growth monitoring in HT29‐xenografted Balb/c nude mice. (B) Time‐dependent body weight monitoring of the mice. (C) HT29 cell migration analysis. (D) HT29 cell proliferation analysis. Cntl, control (PBS). Arrows indicated I.V. injection (A, B). Avastin (M.W., 149 kDa) and 14‐MAP (M.W., 1.4 kDa) were treated as 0.5 mg·kg^−1^ and 0.5 μg·kg^−1^, respectively (A, B), and 5 ng·mL^−1^ (approx. 34 pm) and 50 pg·mL^−1^ (approx. 35 pm), respectively (C, D). *P < 0.05; **P < 0.01 *vs* control. Statistical analysis was conducted using the Student's *t*‐test and nonparametric Kruskal–Wallis test. All mean data were represented with standard error (SEM). Five mice per group were used in the experiment.

Furthermore, we investigated the effect of the antiangiogenic peptide targeting BK in cancer (Fig. 4). The low dose of 14‐MAP (0.5 μg·kg^−1^) considered the working concentration *in vitro* was given to human colon cancer cell (HT29)‐xenograft nude mice by intravenous injection (Fig. 4A,B). The effect of the peptide on reduced tumor growth compared to control was monitored and found to be less than the effect of Avastin, a well‐known anticancer agent targeting VEGF (Fig. 4A). The working dose of Avastin in our study was determined to be 0.5 mg·kg^−1^, which, although not statistically significantly different from other doses, demonstrated the highest efficacy within the tested range of 0.05–5 mg·kg^−1^ (Fig. S5A). The reduction of tumor volume was observed by co‐treatment of 14‐MAP with Avastin (*P* < 0.05–0.01) (Fig. 4A). No treatment induced any change in body weight in the mice (Fig. 4B). However, the pattern of the change in the treatment period showed that the combination of 14‐MAP with Avastin was similar to the control, and 14‐MAP only and Avastin only were slightly less than control at the latter half period (Fig. 4B). Besides, the working dose of 14‐MAP was not toxic to mice (Table S1, Fig. S5B).

Meanwhile, the effect of 14‐MAP on colon cancer cell migration and proliferation was investigated (Fig. 4C,D). The treatment of 14‐MAP (50 pg·mL^−1^), Avastin (5 ng·mL^−1^), or the combination of both showed no significant differences compared to control on cancer cell migration and cancer cell proliferation up to 48 h, but a slightly inhibited pattern was monitored after 14‐MAP treatment rather than the combination treatment on both cancer cell functions (Fig. 4C,D). Moreover, this 14‐MAP effect on cancer cells was confirmed in the results of colony formation for a longer period (over a week) (Fig. S6). In the human colon cancer cells (HT29) and human breast cancer cells (MCF‐7), the treatment of 14‐MAP, Avastin, or the combination of both did not induce any significant difference compared to control (Fig. S6A,B).

## Discussion

Previously, we identified the 14‐MAP sequence located at the second loop between the first and second α‐helices (H1–H2) of the N‐terminal 14K buffalo prolactin (buPRL) and confirmed the antiangiogenic activity of the synthesized peptide through both *in vitro* and *ex vivo* studies [9]. The antiangiogenic properties of the 14‐MAP sequence were validated by observing its significant inhibition of endothelial cell (EC) migration, nitric oxide (NO) production, and chick vessel development in comparison with negative controls, including a scrambled peptide (Scr) and a phosphate‐buffered saline (PBS) control (Figs 1A,B and 2A, Fig. S1).

14‐MAP exhibited characteristics similar to its parent protein, N‐terminal 14K buPRL, notably its potent activity at extremely low concentrations (Figs 1, 2, 3, 4, Fig. S2A). Two key properties were observed: (a) effective function at picomolar concentrations and (b) antiangiogenic activity linked to VEGF‐ and BK‐dependent eNO production (Fig. 2A). Our previous studies corroborate the low‐concentration efficacy, suggesting a possible interaction with high‐affinity binding partners, although these were not identified in the current study. Similar antiangiogenic effects at low concentrations have been recorded for other protein and peptide candidates, like secreted frizzle‐related protein 4, a Wnt binding partner, indicating a broader context for low‐dose activity [32]. Moreover, small peptides, consisting of 3–7 amino acid residues, derived from human PRL potently inhibited angiogenesis and tumor growth (150 pm as IC_50_) [42]. Just three residues, H46‐G47‐R48 of vasoinhibin determined the antiangiogenic function [42]. They draw parallels with the Q46‐G47‐K48 sequence of 14‐MAP derived from buPRL, noting that Q is a conservative substitution of H, R, and K, positively charged amino acid residues. This comparative analysis suggests that the QGK motif of 14‐MAP can be a critical part on antiangiogenic function. Recently, the peptide therapeutics market has been gaining importance [43, 44]. Indeed, 7.2% of total drugs (15/208) approved by the FDA in the last few years (2015–2019) are peptide‐based drugs [45]. Among the peptide‐based drugs, small or medium‐sized peptide drugs such as Macimorelin (Macrilen®, 3‐mer), Plecanatide (Macrilen®, 16‐mer), Afamelanotide (Scenesse®, 13‐mer), etc. account for more than half [45]. The development of peptide‐based drugs has been of interest because of the advantages of size, easy delivery, specificity, and being biologic‐based [43]. In a similar line of thought, we anticipate that 14‐MAP will be a promising candidate for the peptide therapeutics market.

Our current study focused on elucidating the mechanisms by which 14‐MAP exerts its antiangiogenic effects through two well‐established pathways: the VEGF‐ and BK‐mediated eNOS pathways [20, 21]. We found that the peptide antagonistically affected both pathways, inhibiting NO‐mediated endothelial cell functions, although 14‐MAP's activity was more pronounced in the BK‐dependent angiogenic processes compared to VEGF‐dependent ones (Figs 1C–G and 2A). This differential activity suggests that 14‐MAP might preferentially target mature vessels, such as small arteries within the tumor microenvironment [46]. Furthermore, 14‐MAP demonstrated significant interference in the BK/B2R pathway over the Des‐Arg9‐BK/B1R pathway in endothelial cells (Fig. 3). The interference of the antiangiogenic PRL fragments, and vasoinhibins on BK‐induced vascular actions‐NO production via B2R was reported [47]. The interference of the 14‐MAP‐mediated BK‐receptor pathway requires to identification of the antagonistic route with more studies. Furthermore, we demonstrated that 14‐MAP inhibited SpNO‐ and SC‐induced NO production/EC migration of the eNOS/NO/sGC/cGMP/PKG pathway, which happens in the early stage of angiogenesis (Fig. 2). However, it remains unclear, how 14‐MAP interacts with downstream components of cGMP signaling. YC‐1 is an exogenous activator of soluble guanylate cyclase (sGC) that enhances the enzyme's sensitivity to NO, thus amplifying cGMP production. While YC‐1 itself is not a direct NO donor, its activation of sGC can indirectly influence NO levels by promoting feedback mechanisms where elevated cGMP levels enhance eNOS activity and thus NO production. This effect underscores the tight regulation within the NO/sGC/cGMP signaling axis. Given that 14‐MAP did not impair NO levels in conditions where sGC was activated by YC‐1 (Fig. 2B), it suggests that 14‐MAP's inhibitory actions may target upstream elements of the NO signaling cascade rather than sGC directly or the cGMP levels downstream. Specifically, 14‐MAP may interfere with pathways that increase NO production up to the point of sGC activation. This is consistent with our observations of inhibited NO production with SpNO and SC, but not with agents acting post‐NO synthesis such as YC‐1 and 8Br‐cGMP.

The potential therapeutic effects of a BK‐ and eNOS‐dependent antiangiogenic peptide like 14‐MAP in cancer therapy were evaluated (Fig. 4). The combination of 14‐MAP at the low dose (0.5 μg·kg^−1^), not toxic to mice (Table S1, Fig. S5), with the low dose of Avastin (0.5 mg·kg^−1^) led to significantly reduction in tumor growth in human colon cancer‐xenografted to nude mice (Fig. 4A). The statistical difference was monitored between the control and the treatment of 14‐MAP with Avastin (Fig. 4A). The statistical analyses emphasized the combined treatment's efficacy over 14‐MAP alone. Considering that 14‐MAP exhibited no specific targeting of cancer cells (Fig. 4C,D, Figs S5 and S6), its anti‐angiogenic effect likely stems from its interference with BK signaling in endothelial cells. Contemporary antiangiogenic therapies face challenges due to emerging resistance mechanisms, necessitating a shift from VEGF‐dependent to VEGF‐independent angiogenesis targets [6]. Although the reduction in tumor progression by 14‐MAP was less than anticipated, its low dose in combination with Avastin reduced the tumor volume. This combined approach may address the limitations inherent in VEGF‐targeted therapies. However, to fully understand the mechanisms underlying these effects, additional studies are necessary to confirm the role of 14‐MAP's BK‐/eNOS‐dependent antiangiogenic action in tumor reduction.

Bajou *et al*. [48] suggested that N‐terminal 16 K human PRL could serve as an anticancer agent by inducing fibrinolysis and inhibiting angiogenesis via its interaction with plasminogen activator inhibitor‐1 (PAI‐1). Their study confirmed plasminogen activator inhibitor‐1 (PAI‐1) as the direct binding partner of 16K PRL (amino acid residue 176–228 and 244–289, as binding site 1 and 2, respectively) and demonstrated that 16K PRL antagonized PAI‐1 complex (PAI‐1/uPA/uPAR)‐induced thromboembolism and angiogenesis [48]. We do not perceive that 14‐MAP directly binds to PAI‐1 because the 14‐MAP sequence maintains a distance from the two binding sites of 16K PRL. Rather, the 14‐MAP sequence is part of the antiangiogenic domain of vasoinhibin [49]. Recently, Robles *et al*. [49] reported the antiangiogenic domain of vasoinhibin (1–79 residues) derived from the N‐terminal human PRL fragment. The antiangiogenic mechanism of 14‐MAP is probably close to that of the domain of vasoinhibin. BK, widely expressed in vascular tissue, induces pathophysiological processes including angiogenesis, inflammation, and thromboembolism [10, 11, 12]. BK's role in up‐regulating PAI‐1 mRNA and protein through NO‐mediated processes in endothelial cells aligns with this antiangiogenic [12].

Given that thromboembolism constitutes a significant adverse effect of traditional antiangiogenic agents like Avastin, the combination of the BK‐dependent antiangiogenic peptide 14‐MAP and VEGF‐dependent agents offers a promising therapeutic strategy for mitigating such effects [50]. We found that the combination of BK‐dependent antiangiogenic peptide (14‐MAP) and VEGF‐dependent antiangiogenic agent (Avastin) was the most efficient in reducing tumor growth *in vivo*. Our BK‐dependent antiangiogenic peptide can be useful by minimizing the adverse effects of traditional VEGF‐dependent antiangiogenic agents in cancer therapy. Therefore, our study supports the development of multidrug therapeutics for anticancer therapies.

Further, the antiangiogenic properties of 14‐MAP need in‐depth investigations into its relations with other hallmarks of cancer. Questions to be addressed are: is the 14‐MAP efficient enough to target other forms of angiogenesis like intussusceptive angiogenesis, vascular mimicry, or co‐option? Since tumor‐vasculature depends on the cross‐talk among stakeholders in tumor microenvironments like immune cells, cancer stem cells, macrophages, and fibroblasts, understanding cellular and biochemical talks among all stakeholders with 14‐MAP sensitized tumor cells is critical for unraveling the mechanism of 14‐MAP dependent inhibition of tumor angiogenesis [51, 52, 53]. We envisage the further direction of research in 14‐MAP‐based inhibition of tumor angiogenesis warrants support from omics‐based interactome studies of the tumor microenvironment.

## Conflict of interest

The authors declare no conflict of interest.

### Peer review

The peer review history for this article is available at https://www.webofscience.com/api/gateway/wos/peer‐review/10.1002/2211‐5463.13895.

## Author contributions

JL, SM, PK, KM and SC contributed to conceptualization. SM, PK, SN, JL and SC contributed to methodology. JL, SM, KM and SC contributed to formal analysis. JL, PK, SN, SHP, LS, J‐SC, HJL, KM and SC contributed to investigation. J‐SC and KM contributed to resources. JL contributed to data curation. JL, PK, HJL and SC contributed to writing—original draft preparation. JL, PK, HJL and SC contributed to writing—review and editing. JL and PK contributed to visualization. HJL and SC contributed to supervision. JL, HJL and SC contributed to project administration. JL, HJL and SC contributed to funding acquisition. All authors have read and agreed to the published version of the manuscript.

## Supporting information


**Fig. S1.** No inhibitory effect of Scr on chick vessel development.
**Fig. S2.** Working concentration of 14‐MAP in EC.
**Fig. S3.** 14‐MAP effect on BK‐ and VEGF‐induced EC functions.
**Fig. S4.** Diagram of BK‐BKR pathways.
**Fig. S5.** *In vivo* analysis.
**Fig. S6.** Effect of 14‐MAP on colony formation of cancer cells.
**Table S1.** 14‐MAP toxicity in ICR mice.

## Data Availability

Data is contained within the article or Supporting Information, and the raw data supporting the conclusions of this article will be made available by the authors, without undue reservation.
