# Supplementary material for: 14K prolactin derived 14‐mer antiangiogenic peptide targets bradykinin‐/nitric oxide‐cGMP‐dependent angiogenesis
Source: FEBS Open Bio. 2024 Sep 23;14(12):2072–85. doi: 10.1002/2211-5463.13895 (PMC11609586; doi:10.1002/2211-5463.13895)
Supplement: Supplementary file 1 — Fig. S1. No inhibitory effect of Scr on chick vessel development. Fig. S2. Working concentration of 14‐MAP in EC. Fig. S3. 14‐MAP effect on BK‐ and VEGF‐induced EC functions. Fig. S4. Diagram of BK‐BKR pathways. Fig. S5. In vivo analysis. Fig. S6. Effect of 14‐MAP on colony formation of cancer cells. Table S1. 14‐MAP toxicity in ICR mice. [file FEB4-14-2072-s001.docx]

Supplementary Information

**Figure S1.**


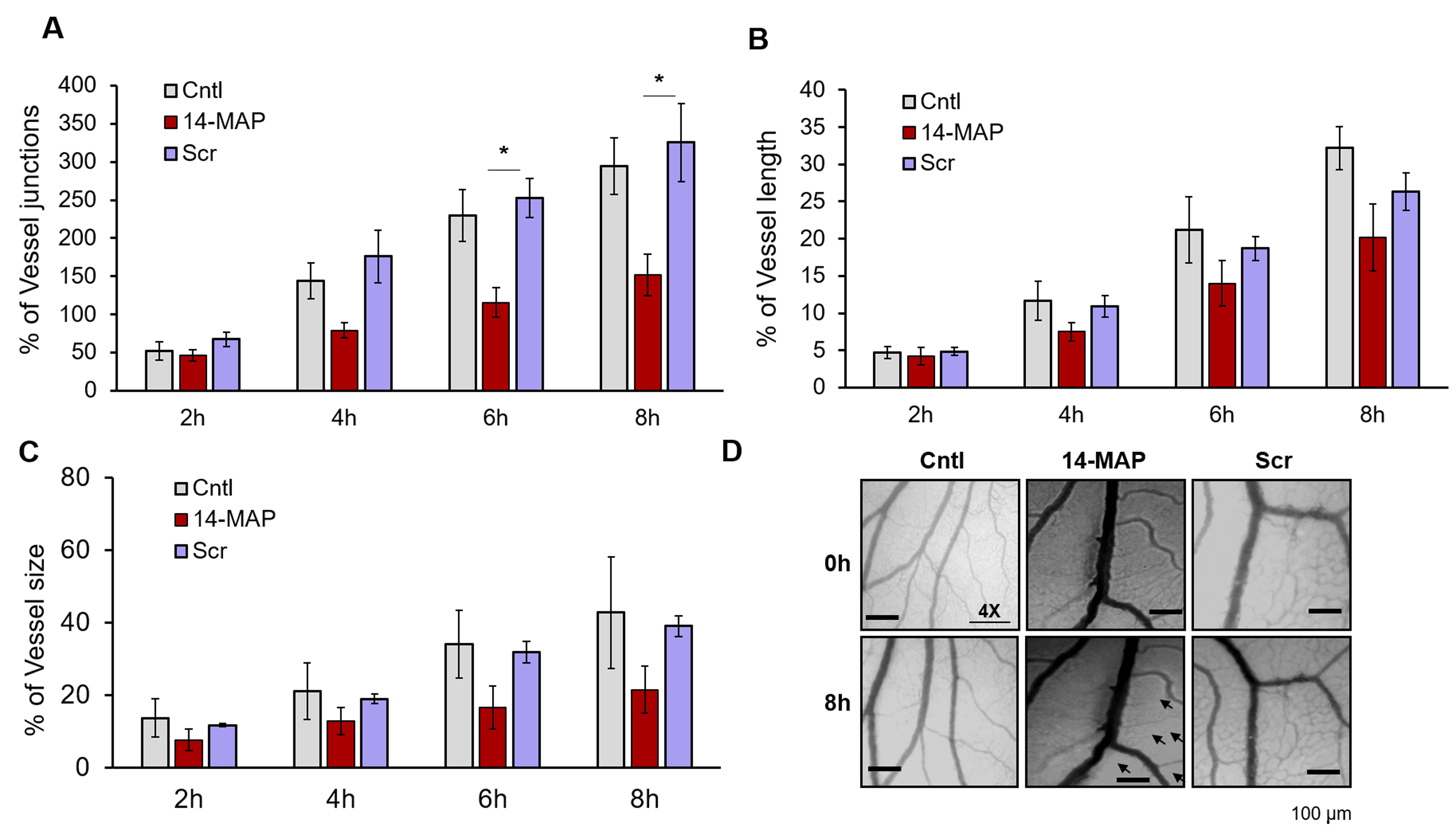


**Figure S1. No inhibitory effect of Scr on chick vessel development.** Analysis of vessel junction (**A**), length (**B**) and size (**C**) by CAM assay. **D**, Pictures of vessel development. 10 pg/mL was used as the concentration of 14-MAP and Scr peptide. Scale 100 µm. Cntl, control; Scr, scramble (GSQCAAGTMNLKIF). *, *P* < 0.05, arrows represent indicate the deteriorating vascular network. Statistical analysis was conducted using Tukey’s or Dunnett’s test in conjunction with the one-way ANOVA test or Dunnett’s T3 test in conjunction with Welch's one-way ANOVA. All mean data were represented with standard error (SEM). Each experiment represents three independent biological experiments.

**Figure S2.**


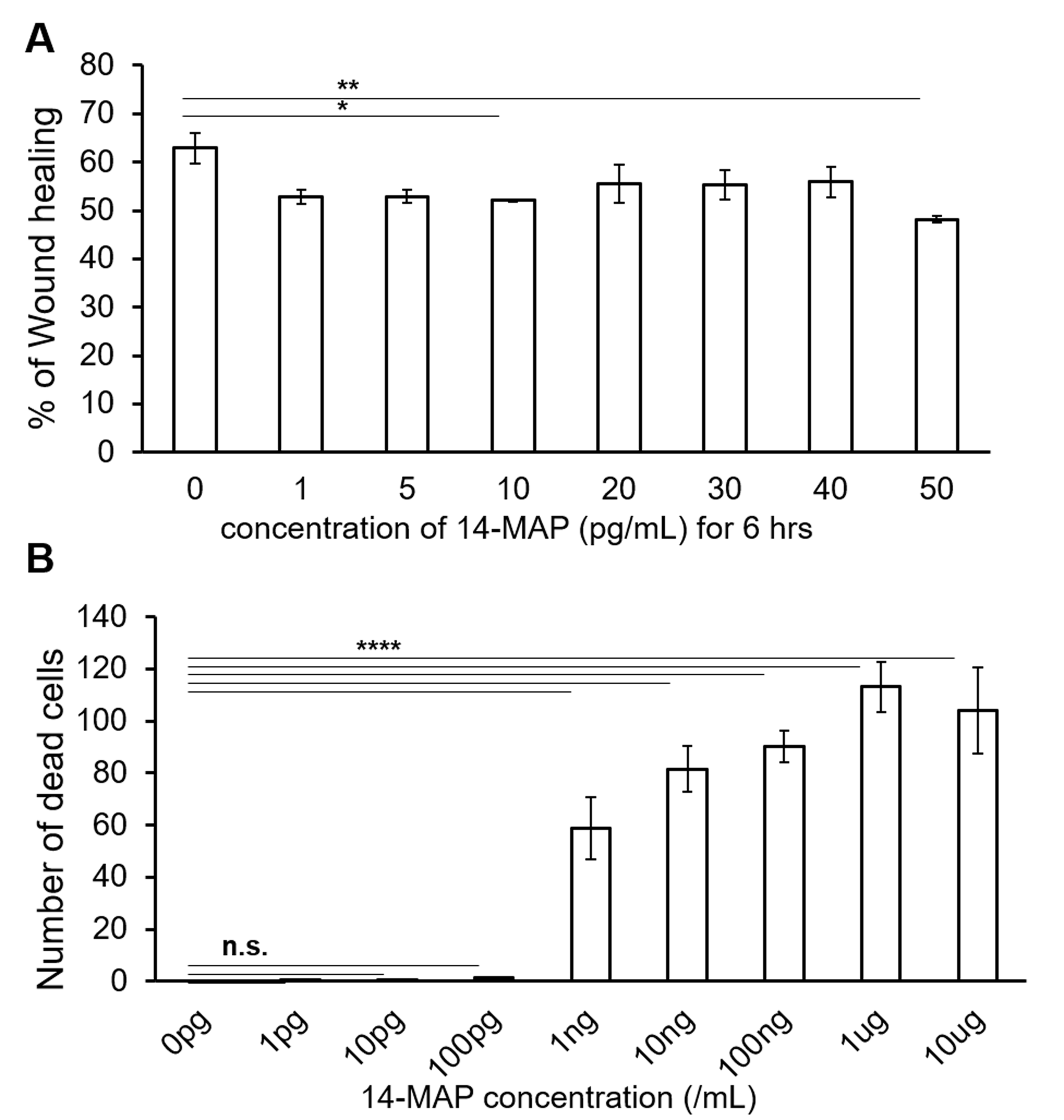


**Figure S2. Working concentration of 14-MAP in EC. A**, Cell migration analysis and **B**, cytotoxicity analysis after treatment with 14-MAP peptide (M.W. 1.4 kDa). n.s., no significant; *, *P* < 0.05; **, *P* < 0.01; ****, *P* < 0.0001. Statistical analysis was conducted using Tukey’s or Dunnett’s test in conjunction with the one-way ANOVA test or Dunnett’s T3 test in conjunction with Welch's one-way ANOVA. All mean data were represented with standard error (SEM). Each experiment represents three independent biological experiments.

**Figure S3.**


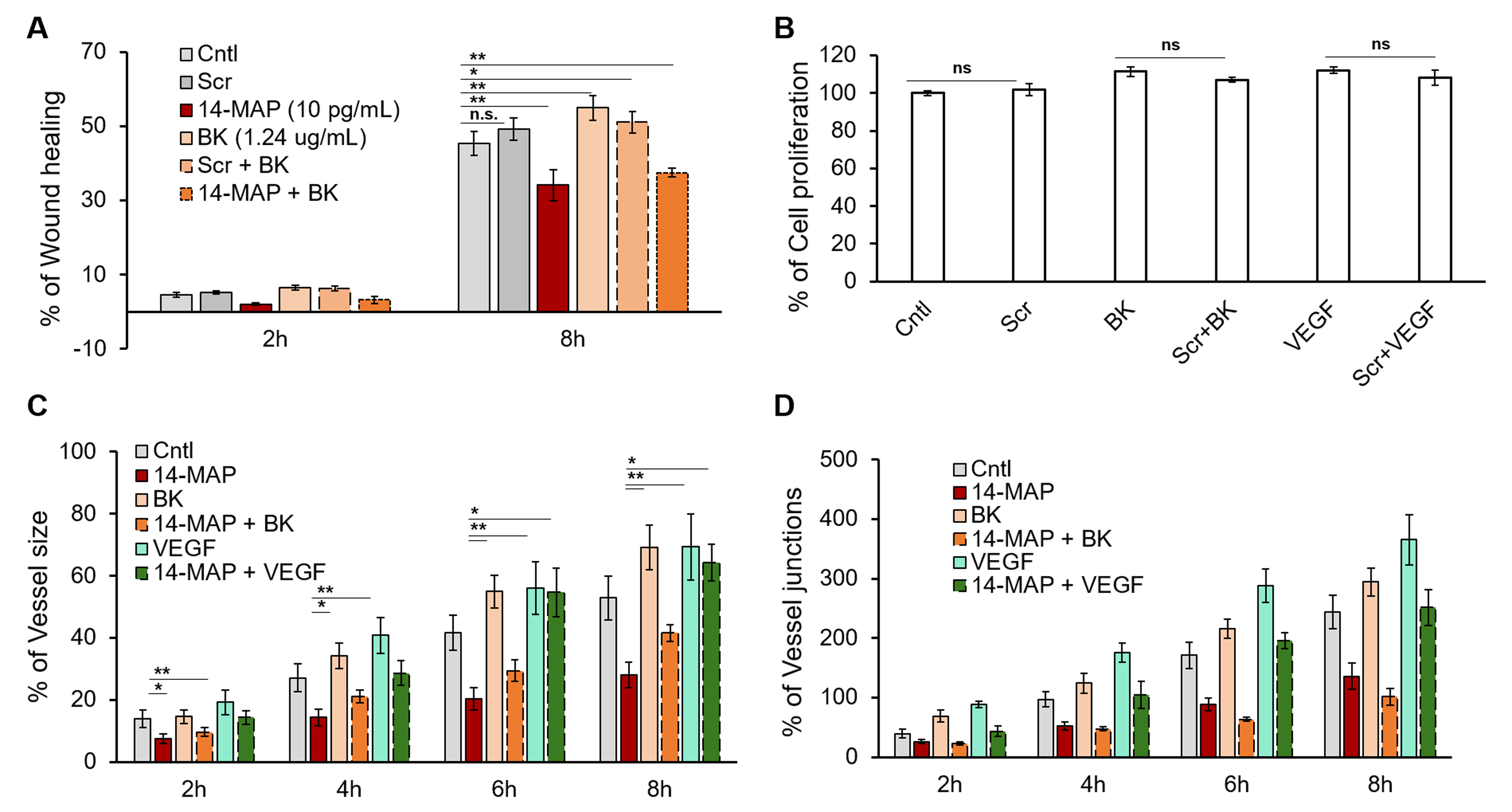


**Figure S3. 14-MAP effect on BK- and VEGF-induced EC functions. A**, 14-MAP effect on BK-induced cell migration. **B**, Scr effect on BK- and VEGF-increased cell proliferation. **C** and **D**, 14-MAP effect on BK- and VEGF-improved chick vessel size and junction, respectively. Cntl, control; Scr, scramble (GSQCAAGTMNKIF). n.s., no significant; *, *P* < 0.05; **, *P* < 0.01. Statistical analysis was conducted using Tukey’s or Dunnett’s test in conjunction with the one-way ANOVA test or Dunnett’s T3 test in conjunction with Welch's one-way ANOVA. All mean data were represented with standard error (SEM). Each experiment represents three independent biological experiments.

**Figure S4**


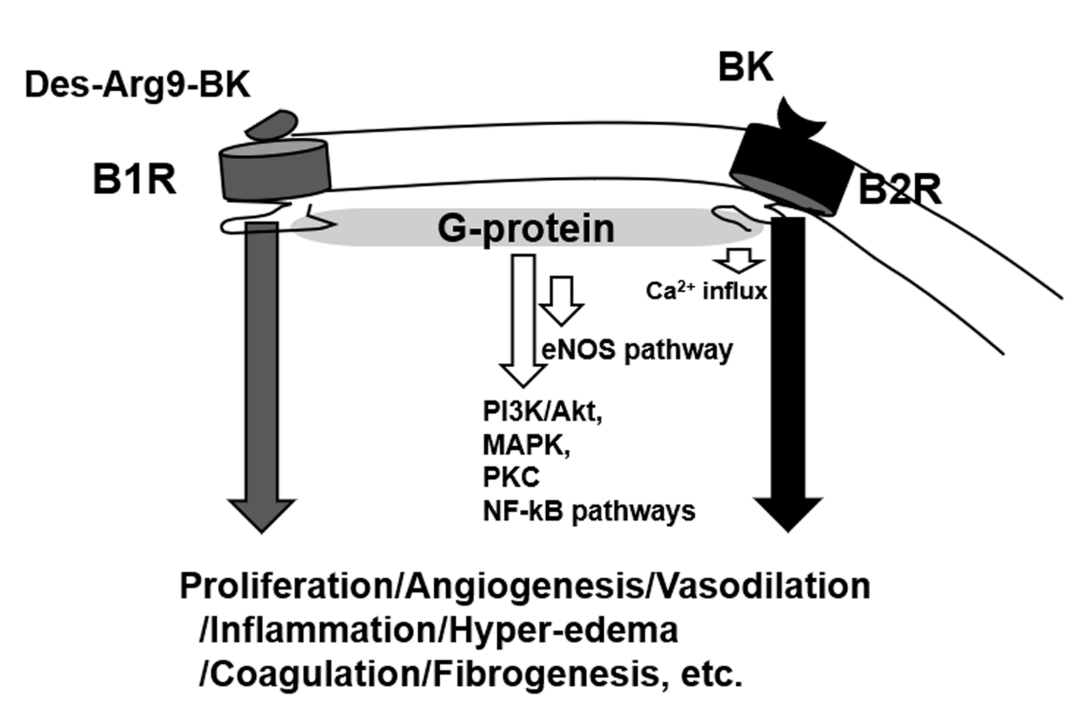


**Figure S4.** Diagram of BK-BKR pathways.

**Figure S5.**


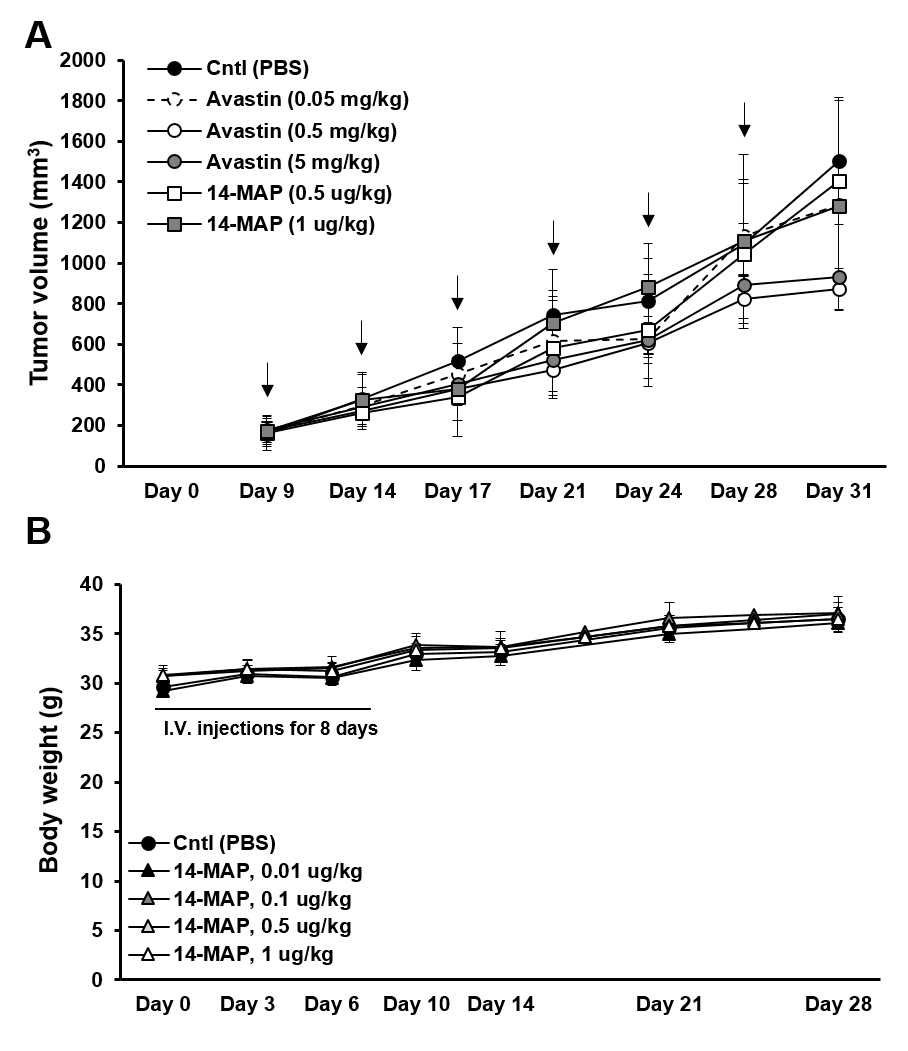


**Figure S5. *In vivo* analysis. A**, Dose determination of 14-MAP (M.W., 1.4 kDa) and Avastin (M.W., 149 kDa) in Balb/c nude mice. **B**, Toxicity analysis (body weight) after 14-MAP treatment to ICR mice. Cntl, control. Arrows indicated I.V. injection. Statistical analysis was conducted using the Student’s *t*-test and nonparametric Kruskal-Wallis test. All mean data were represented with standard error (SEM). Five mice per group were used in the experiment.

**Figure S6.**


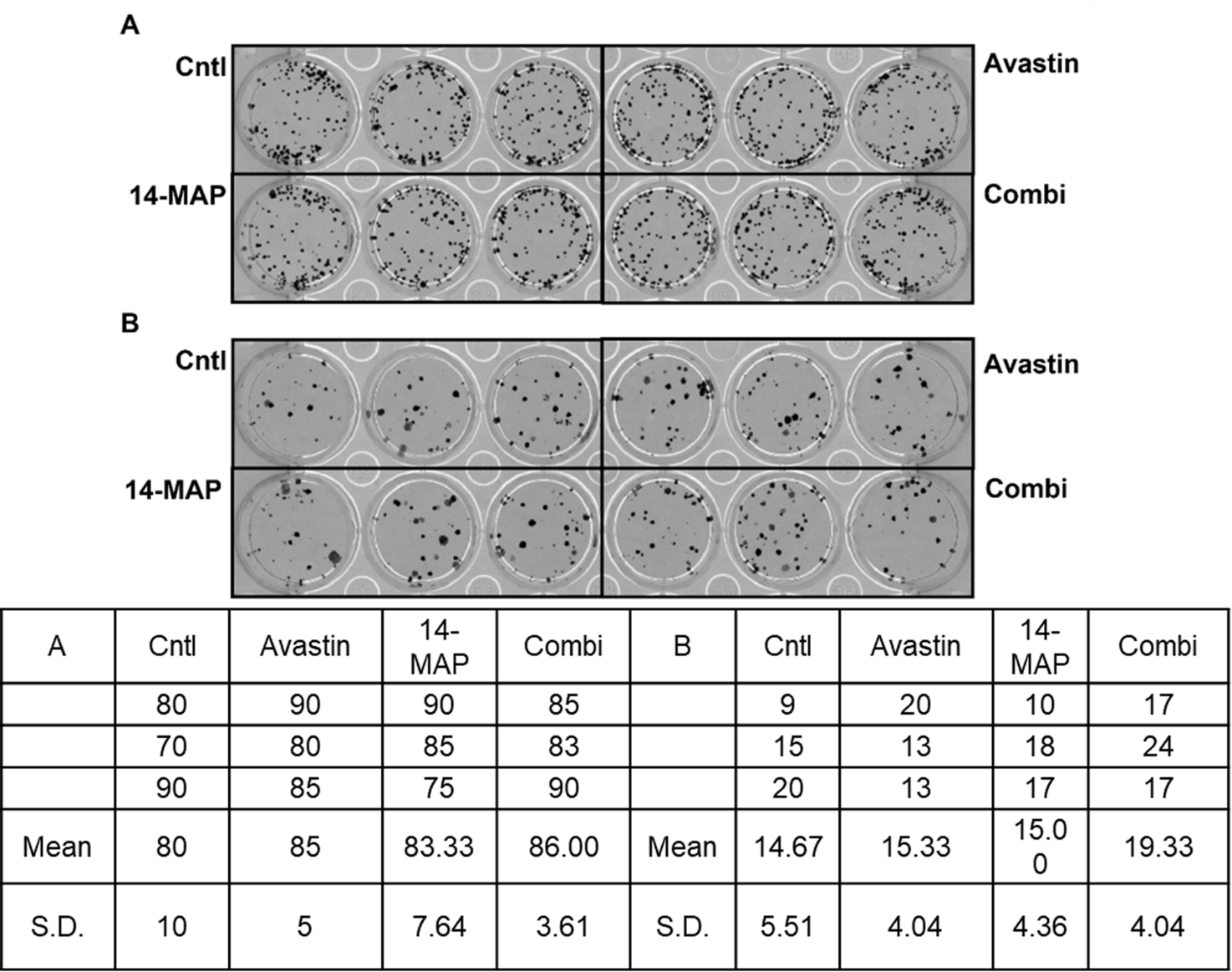


**Figure S6. Effect of 14-MAP on colony formation of cancer cells. A**, HT29 cells and **B** MCF-7 cells. Cntl, control, PBS; Avastin, 5 ng/mL (approx. 34 pM); 14-MAP, 50 pg/mL (approx. 35 pM); combi, combination of Avastin and 14-MAP. Statistical analysis was conducted using Tukey’s or Dunnett’s test in conjunction with the one-way ANOVA test or Dunnett’s T3 test in conjunction with Welch's one-way ANOVA. All mean data were represented with standard error (SEM). Each experiment represents three independent biological experiments.

**Supplementary Table 1. 14-MAP toxicity in ICR mice.**

| **Group** | **Survival Rate (%)** |
| --- | --- |
| **Control (PBS)** | **100** |
| **14-MAP, 0.01 µg/kg** | **100** |
| **14-MAP, 0.1 µg/kg** | **100** |
| **14-MAP, 0.5 µg/kg** | **100** |
| **14-MAP, 1 µg/kg** | **100** |
